# Comparative effects of topiramate and naltrexone on neural activity during anticipatory anxiety in individuals with alcohol use disorder

**DOI:** 10.1093/alcalc/agae078

**Published:** 2024-11-18

**Authors:** Gezelle Dali, Warren Logge, Henry R Kranzler, Tristan Hurzeler, Hugh Gallagher, Paul S Haber, Kirsten C Morley

**Affiliations:** Specialty of Addiction Medicine, Sydney Medical School, Faculty of Medicine and Health, University of Sydney, Sydney, NSW 2050, Australia; Edith Collins Centre for Translational Research (Alcohol, Drugs & Toxicology), Royal Prince Alfred Hospital, Sydney Local Health District, Sydney, NSW 2050, Australia; Specialty of Addiction Medicine, Sydney Medical School, Faculty of Medicine and Health, University of Sydney, Sydney, NSW 2050, Australia; Edith Collins Centre for Translational Research (Alcohol, Drugs & Toxicology), Royal Prince Alfred Hospital, Sydney Local Health District, Sydney, NSW 2050, Australia; Center for Studies of Addiction, University of Pennsylvania Perelman School of Medicine and Crescenz Veterans Affairs Medical Center, Philadelphia, PA 19104, United States; Specialty of Addiction Medicine, Sydney Medical School, Faculty of Medicine and Health, University of Sydney, Sydney, NSW 2050, Australia; Specialty of Addiction Medicine, Sydney Medical School, Faculty of Medicine and Health, University of Sydney, Sydney, NSW 2050, Australia; Specialty of Addiction Medicine, Sydney Medical School, Faculty of Medicine and Health, University of Sydney, Sydney, NSW 2050, Australia; Edith Collins Centre for Translational Research (Alcohol, Drugs & Toxicology), Royal Prince Alfred Hospital, Sydney Local Health District, Sydney, NSW 2050, Australia; Specialty of Addiction Medicine, Sydney Medical School, Faculty of Medicine and Health, University of Sydney, Sydney, NSW 2050, Australia; Edith Collins Centre for Translational Research (Alcohol, Drugs & Toxicology), Royal Prince Alfred Hospital, Sydney Local Health District, Sydney, NSW 2050, Australia

**Keywords:** alcohol use disorder, topiramate, naltrexone, anxiety, fMRI

## Abstract

Topiramate has been found to be effective in reducing alcohol use and may also attenuate anxiety severity in patients with alcohol use disorder (AUD). This study compared the neural response of treatment-seeking patients with AUD on either topiramate or naltrexone during an anticipatory anxiety task. Participants were 42 patients with AUD who were randomized to receive either topiramate (*n =* 23; titrated dose up to 200 mg/day) or naltrexone (*n* = 19; 50 mg/day) for 12-weeks as part of a larger randomized controlled trial. Following 6 weeks of treatment, participants completed an anticipatory anxiety task during a functional magnetic resonance imaging (fMRI) session. The task presented a series of high-threat and low-threat stimuli followed by an unpleasant or pleasant image, respectively. Primary whole-brain analyses revealed no significant differences in neural activation between the topiramate and naltrexone groups. Deactivation for safe cues relative to threat cues was observed within the precuneus, inferior parietal lobule and the cingulate gyrus. In the precentral and middle frontal gyri, threat cues elicited greater activation. Exploratory analyses revealed an effect of change in anxiety from baseline to week 6, with a greater reduction associated with a reduced response to threat cues relative to safe cues in the cuneus and lingual gyrus. The current study is the first to examine and compare neural activation during anticipatory anxiety in treatment-seeking individuals on topiramate and naltrexone. This preliminary research contributes to our understanding of the therapeutic mechanisms of these alcohol pharmacotherapies.

## Teaser

**Short Summary:** This study compared the neural response of patients with AUD treated with topiramate or naltrexone during an anticipatory anxiety task. No significant differences in neural activation were found between the groups, however, reductions in anxiety were linked to decreased threat response.

## Introduction

Alcohol consumption is associated with extensive physical and mental health consequences and is implicated in 5.1% of the global burden of disease (World Health Organization, 2014). Problematic alcohol use can result in alcohol use disorder (AUD), a chronic relapsing disorder characterized by compulsive alcohol-seeking and consumption despite negative consequences (American Psychiatric Association 2013). Chronic heavy alcohol consumption has been found to result in alterations to several neurotransmitter systems including γ-aminobutyric acid (GABA), glutamate, and central dopamine and noradrenaline activity (Vengeliene et al. 2008; Haass-Koffler et al. 2018). First line medications for treating AUD, such as naltrexone and acamprosate, typically target these systems with varying levels of efficacy in reducing consumption or promoting abstinence. Topiramate is an anticonvulsant medication that is used off-label in treating AUD. Topiramate appears to inhibit dopamine release in the mesocorticolimbic dopamine pathways by enhancing GABA neurotransmission (White et al. 2000) and/or inhibiting glutamatergic neurotransmission (via AMPHA/kainate receptors) (Ängehagen et al. 2005). Meta-analyses have demonstrated the potential utility of topiramate in reducing alcohol use and craving, relative to placebo (Blodgett et al. 2014; Fluyau et al. 2023). More recent evidence indicates that topiramate has efficacy that is at least comparable to standard care (naltrexone), with greater improvements on some outcomes (Morley et al. 2024). A greater understanding of the therapeutic mechanisms is required, however, to facilitate the development of personalized treatment strategies.

Topiramate may be efficacious in treating AUD, at least in part, by reducing anxiety related to alcohol withdrawal (Junqueira-Ayres et al. 2017). Indeed, topiramate has been found to reduce anxiety severity in patients with AUD (Paparrigopoulos et al. 2011; Martinotti et al. 2014). It has been proposed that topiramate normalizes the activity of reward circuits in the brain, thereby restoring a normal stress response in patients with AUD (Manhapra et al. 2019). This potential mechanism of action is important to consider as AUD frequently co-occurs with anxiety disorders or heightened levels of anxiety (Castillo-Carniglia et al. 2019). Indeed, individuals with AUD experience approximately twice the risk of an anxiety disorder compared to those without AUD (Lai et al. 2015). Moreover, severity of anxiety has been purported to predict early relapse and worse treatment outcomes for individuals with AUD (Kushner et al. 2000; Schellekens et al. 2015). The tension reduction hypothesis has been proposed to explain the relationship between anxiety and AUD (Khantzian 1997). That is, individuals with AUD who experience anxiety consume alcohol to relieve tension and develop an over-reliance on alcohol to relieve negative affect in the future. It has also been suggested that elevated anxiety in the long-term may result from heavy, prolonged alcohol consumption (Torvik et al. 2019).

Functional neuroimaging studies have examined neural responses during tasks evoking negative affect in AUD samples. For example, in an anticipatory anxiety task, individuals with AUD have been shown to display hypoactivation in response to high-threat stimuli relative to low-threat stimuli in the rostral anterior cingulate cortex (ACC), ventromedial prefrontal cortex and precuneus/posterior cingulate cortex (PCC) (Yang et al. 2013), relative to controls. These regions have been posited to be involved in the evaluation and expression of negative emotion, including the regulation and inhibition of stimulus-induced anxiety (Yang et al. 2013). Other studies comparing participants with AUD to healthy controls have implicated the medial frontal gyrus (Padula et al. 2015), amygdala and insula in affect reactivity, such that greater severity of alcohol dependence was associated with lower activation and reduced connectivity (O'daly et al. 2012). These regions have similarly been found to be associated with affective and fear processing. The insula, in particular, is involved in recognizing the emotional significance of stimuli and generating affective responses. Connectivity between the insula and prefrontal areas, such as the ACC, is suggested to subserve emotional processing (Klumpp et al. 2012).

Functional neuroimaging studies examining the effect of pharmacotherapy on anxiety responses in AUD patients are sparse. A previous pharmaco-fMRI study employed an anticipatory anxiety task in patients with AUD on either prazosin (an alpha-1 adrenergic receptor blocker) or placebo as part of a broader randomized controlled trial (Wilcox et al. 2020). The study found that prazosin did not modulate neural activation during high-threat stimuli, though greater deactivation was found to be associated with worse long-term treatment outcomes. Existing work has also demonstrated an effect of nalmefene – a functional opioid system antagonist – on emotional processing in patients with AUD. Using a cross-over design, Vollstädt-Klein et al. (2019) found greater activity in regions implicated in social interaction and empathy such as the supramarginal gyrus, angular gyrus and putamen in response to emotional picture sets when participants were administered nalmefene compared to placebo. More recently, we demonstrated that baclofen – a selective GABA_B_ receptor agonist – attenuated insula activity in an anticipatory task in individuals with AUD, relative to placebo (Morley et al. 2021). This finding suggests that the modulation of responses to high-threat cues may be mediated by GABA receptors, which may be a mechanism by which baclofen exerts treatment effects on AUD. Given the potential anti-anxiolytic effect of topiramate, it is thus of interest to determine whether topiramate similarly attenuates neural activity during anticipation of high-threat stimuli.

No study thus far has yet examined the role of topiramate relative to standard care pharmacotherapy on fMRI reactivity to anticipatory anxiety. As part of a clinical trial, this study compared the anticipatory neural activity of patients with AUD undergoing treatment with topiramate or naltrexone. Naltrexone was selected as an active control in the wider trial because it is an approved and widely available pharmacotherapy for AUD. Naltrexone is a μ-opioid receptor antagonist that reduces dopamine levels in the NAc by attenuating the release of endogenous opioids following alcohol consumption (Maisel et al. 2013). Previously, it has been proposed that topiramate may have broader efficacy than the anti-craving effects of naltrexone by also reducing anxiety and dysphoria related to the withdrawal of alcohol (Baltieri et al. 2008). Based on fMRI results from prior anticipatory anxiety and affect reactivity studies, we hypothesized that patients with AUD receiving topiramate would demonstrate significantly different brain activation in neural regions that have been implicated in anxiety and fear conditioning (e.g. insula, precuneus) relative to patients receiving naltrexone.

## Materials and methods

### Overview

The current study was conducted as part of a broader double-blind randomized trial comparing the therapeutic and cost-effectiveness of topiramate and an active control (naltrexone) in improving treatment outcomes for individuals with AUD (Morley et al. 2018). The study was approved by the Sydney Local Health District Ethics Review Committee (X16–0231 and HREC/16/RPAH/283). The study involved an off-label use of a registered medication (topiramate) in Australia and approval was given under the Clinical Trial Notification Scheme of the Therapeutic Goods Administration (2013/0060). Participants were randomized to receive either topiramate (titrated dose up to 200 mg/day) or naltrexone (50 mg/day) for 12 weeks. Randomization was provided by an independent service at the Clinical Trials Centre of the National Health and Medical Research Council in Australia using minimization stratified by *GRIK1* genotype, *OPRM1* genotype and the concurrent use of antidepressants. The topiramate dose remained stable from the 6-week visit until the 12-week visit. Scanning occurred once participants had received at least 6 weeks of medication in the main study (i.e. 6–8 weeks from the first dose of study medication) in order to ensure titration of topiramate was complete. All participants provided written informed consent to be included in the present study.

**Figure 1:**
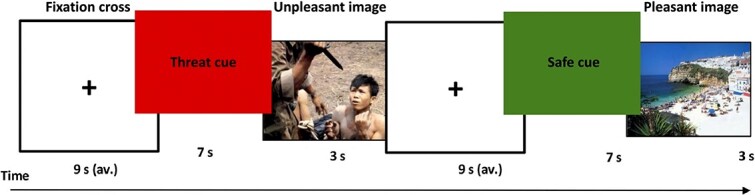
Anticipatory anxiety task.

### Participants

Treatment-seeking individuals who provided informed consent and were eligible for the larger clinical trial (*N* = 42) were recruited to participate in the current study. Full inclusion and exclusion criteria for the main trial have been reported elsewhere (Morley et al. 2018). Most pertinently, participants were required to have an AUD diagnosis according to the DSM-5; and an average weekly consumption of ≥30 standard drinks for men and ≥ 25 standard drinks for women, with a weekly average of ≥2 heavy drinking days during the month prior to screening. Participants were excluded from the main trial if they presented with an active major psychiatric disorder associated with psychosis, significant suicide risk or impaired cognitive functioning; dependence on any substance other than nicotine; and concurrent use of any psychotropic medication other than antidepressants (excluding tricyclic antidepressants). Additional exclusion criteria for the current sub-study included: (i) History of head trauma or injury causing loss of consciousness, lasting more than 5 minutes or associated with skull fracture or intracranial bleeding or abnormal MRI; (ii) History of seizures; (iii) Presence of metal (unless confirmed by a radiologist to be unproblematic); (iv) Claustrophobia or other medical condition preventing the patient from lying in the MRI for ~1 hour; (v) Vision problems that cannot be corrected with glasses; (vi) Due to scanning site restrictions, body girth >52 inches or a head girth >25 inches or greater than the weight limit as per Southern Radiology; (vii) Individuals suffering from or with a history of stroke and/or stroke-related spasticity; (viii) Individuals who are HIV positive or have advanced alcohol-related liver disease, due to potential neurocognitive deficits, even in otherwise asymptomatic individuals, which could confound the results of fMRI testing; (ix) Individuals who have taken topiramate for AUD and report no treatment response; (x) Urine drug screen positive for recent use of opioids, cocaine, or amphetamines; (xi) Breath alcohol concentration > .00 g% on the day of scanning.

### Procedure

#### Assessments

At baseline and each subsequent appointment, consumption in the preceding 30 days was assessed using the Timeline Follow-back method (Sobell and Sobell 1992) in number of Australian standard drinks (which contain 10 g ethanol). AUD severity was measured using the Alcohol Dependence Scale (ADS; Skinner and Allen 1982). Depression, anxiety and stress levels were assessed using the Depression, Anxiety and Stress Scales (DASS-42) (Lovibond and Lovibond 1995).

### fMRI anticipatory anxiety task

Participants were administered an adapted version of an anticipatory anxiety task published previously (Morley et al. 2021) (Fig. 1). The task presented a series of high-threat (‘threat’) and low-threat (‘safe’) stimuli followed by the presentation of an unpleasant or pleasant image. Participants were instructed that a green screen background accompanied by the word ‘SAFE’ indicated the forthcoming presentation of a positive image. Conversely, a red screen background paired with the word ‘THREAT’ indicated that a negative, aversive image would follow. Images were matched for arousal and valence according to the International Affective Picture System and were presented for 3 s in blocks of three images of the same type (unpleasant or pleasant). Stimuli and block order were randomized across participants, and blocks of the same image type were not presented consecutively. Each condition block was interspersed with a separate arrow response attention task which required participants to indicate the left or right direction of an arrow within a 4 s window. Responses were made using a Lumina MRI-compatible two-button response pad. Following each response, a fixation cross was presented for an average overall trial length of 9 s (jittered 8, 9, 10 s). Participants rated their anxiety and craving for alcohol using an 11-point visual analogue scale (rated from 0 to 10) prior to the scanning session, immediately following the anticipation task and at the conclusion of the scanning session. Initiation of the anticipation task was triggered by an MRI scanner pulse to ensure precise temporal equivalence of stimulus presentation and fMRI data acquisition.

### Image acquisition

MRI data were acquired on a 3-Tesla GE Discovery scanner (GE Healthcare, Milwaukee, Wisconsin, USA) using a 32-channel head coil. A T1-weighted (1-mm^3^ voxel resolution) structural scan was acquired for each participant for screening and registration (TR, 7200 ms; TE, 2.7 ms; 176 sagittal slices; 1 mm thick; no gap; 256 × 256 × 256 matrix). For BOLD acquisition, we acquired 203 echoplanar image volumes comprising 39 axial slices in an ascending interleaved fashion with a voxel resolution of 1.88 × 1.88 × 2 mm (TR, 3000 ms; TE, 30 ms; FA, 90°; FOV, 240 mm; matrix, 128 × 128; acceleration factor, 2; slice gap, 1 mm). Foam pads were used to minimize head movement.

### Image processing

Pre-processing and statistical analyses of functional neuroimaging data were conducted using SPM12 (Wellcome Trust Centre for Neuroimaging, London, UK, www.fil.ion.ucl.ac.uk/spm). Functional images were slice-time corrected to the middle slice and realigned with the first volume. The structural image was co-registered to the mean functional image, segmented and warped to Montreal Neurological Institute (MNI) space. The warp parameters were then used to normalize the resampled functional images (2mm^3^). Images were then smoothed with a Gaussian kernel of 8 mm full-width half maximum to improve sensitivity for group analysis.

### Statistical analysis

Demographic information and clinical characteristics were analysed using the programming language R (R Core Team 2021). Assumptions were tested and non-parametric analyses were computed under violations of normality. Imaging data were analysed at two levels using SPM12 and the Multivariate and Repeated Measures for Neuroimaging (MRM) toolbox. The MRM toolbox affords a multivariate and permutation approach that allows advanced statistical modelling of repeated measures mixed-effects designs using a multivariate form of the general linear model (GLM) employed within MATLAB. In the first level (subject-specific), threat cues and safe cues were modelled in SPM12 as box-car functions convolved with the canonical haemodynamic response. Motion correction parameters (six regressors) and arrow task blocks were also modelled as regressors of no interest within the first-level model. The fixation cross served as an implicit baseline. Individual beta maps for threat and safe cues were entered into a second-level whole-brain multivariate GLM in MRM which compared topiramate and naltrexone groups using a two-way mixed-effects design. As there was a difference in mean ADS score between the groups, ADS was included as a covariate in all analyses. Statistical thresholds were set using permutation-based inference, with 5000 permutations conducted to account for the within- and between-subjects variance in the model while assessing the betas for the two conditions. Whole-brain analyses were corrected using a family-wise error cluster-level inference (pFWEc) at < .05 with a cluster-forming height threshold of *P* = .001, in accordance with recommendations by Eklund et al. (2016).

## Results

### Sample characteristics

Table 1 summarizes the demographic and clinical characteristics of the sample. There were no significant differences between the groups on any baseline or pre-scan measure. The topiramate group, however, had a higher baseline ADS score, *t =* 2.54, *P* = .015.

**Table 1:** Descriptive statistics of sample characteristics at baseline and pre-scan

	Naltrexone (*n* = 19)	Topiramate (*n* = 23)	*P-*value
Age	48.89 (11.40)	44.52 (8.07)	0.169
Sex, n (%) F	9 (47)	7 (30)	0.421
Education, y	14.79 (4.06)	15.43 (3.12)	0.584
Unemployed, n (%)	8 (42)	9 (39)	0.999
Years problem drinking	10.21 (8.47)	14.29 (9.58)	0.157
Anti-depressant use, n (%)	9 (47)	12 (52)	0.999
ADS	17.08 (6.14)	22.27 (6.96)	0.015
DASS-42 depression	15.79 (11.68)	17.04 (9.04)	0.648
DASS-42 anxiety	10.21 (6.83)	11.65 (6.57)	0.183
DASS-42 stress	18.32 (10.76)	19.30 (8.58)	0.742
Baseline drinks per drinking day^a^	11.46 (5.02)	13.51 (6.63)	0.371
Pre-scan drinks per drinking day^b^	4.47 (4.10)	6.29 (4.57)	0.223
Pre-scan treatment days^b^	49.95 (13.14)	47.43 (7.91)	0.879
Pre-scan recent drink, n (%)^c^	6 (32)	9 (39)	0.853
Pre-scan heavy drinking days, (%)^b^^,^^d^	12.70 (23.42)	18.84 (21.12)	0.245

*Note.* Data represent mean (standard deviation) unless noted otherwise. ADS = Alcohol Dependence Scale; DASS-42 = Depression, Anxiety and Stress Scale – 42.

^a^In the 30 days prior to trial enrolment

^b^From baseline to the day before scanning

^c^In the 24 hours prior to scanning

^d^Defined as ≥4 drinks for women, and ≥ 5 drinks for men

### Subjective anxiety and craving

State anxiety and craving were reported using a visual analogue scale pre-scanning session, post-task and post-scanning session (see Table 2 for scores). There were no significant differences in reported anxiety either between the groups *F*(1, 40) = 1.31, *P =* .259, η_p_^2^ = .032, or across timepoints, *F*(1.37, 54.83) = 1.29, *P =* .275, η_p_^2^ = .031, nor an interaction effect, *F*(1.37, 54.83) = 1.96, *P =* .163, η_p_^2^ = .047. Similarly, there were no differences in craving between the groups, *F*(1, 40) = .07, *P =* .798, η_p_^2^ = .002, nor an interaction between the groups and time, *F*(1.80, 72.14) = 2.20, *P =* .123, η_p_^2^ = .052. There was a difference in craving across the timepoints, *F*(1.80, 72.14) = 4.49, *P =* .017, η_p_^2^ = .101, with craving significantly lower post-task than post-scanning session, *t*(40) = 2.84, *P* = .020. In addition to baseline, anxiety symptomology was measured at week 6 and week 12 as part of the broader trial, and no group differences were observed in the sub-sample at week 6, W = 202.5, *P* = .592 (naltrexone, *M* = 3.35, *SD =* 5.51; topiramate, *M* = 3.83, *SD =* 4.74), and week 12, W = 213.5, *P* = .167 (naltrexone, *M* = 3.06, *SD =* 5.06; topiramate, *M* = 4.60, *SD =* 4.95).

**Table 2:** Measures of anxiety and craving before and after scanning.

	Naltrexone (*n* = 19)		Topiramate (*n* = 23)	
	Mean (SD)	Range	Mean (SD)	Range
VAS pre-scan craving	1.11 (1.79)	0–6	1.91 (2.52)	0–8
VAS post-task craving	0.89 (1.59)	0–6	0.61 (1.44)	0–6
VAS post-scan craving	1.53 (1.98)	0–7	1.39 (1.97)	0–7
VAS pre-scan anxiety	1.89 (2.21)	0–7	1.74 (2.09)	0–8
VAS post-task anxiety	2.6\3 (2.61)	0–8	1.30 (1.74)	0–6
VAS post-scan anxiety	1.63 (1.89)	0–7	1.30 (1.89)	0–7

*Note.* Data represent mean (standard deviation). DASS-42 = Depression, Anxiety and Stress Scale – 42. VAS = visual analogue scale.

### Anticipatory anxiety

**Figure 2:**
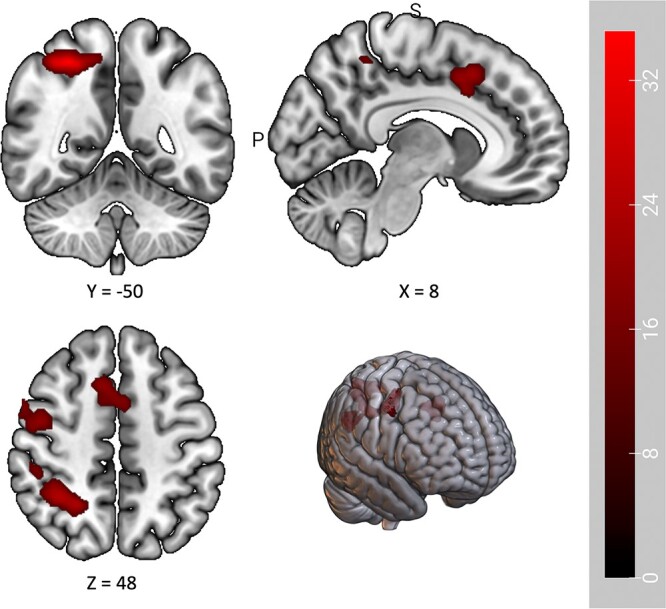
Clusters demonstrating significantly different activation for threat versus safe cues. Brighter colours represent greater activation in response to threat cues. Activation maps presented at *P* < .001 corrected, with the colour bar representing T-scores.

Contrary to expectation, primary whole-brain analyses revealed no significant differences in neural activation between the topiramate and naltrexone groups in response to threat cues versus safe cues. Similarly, there was no overall effect of treatment on neural activation. Across the whole sample, three clusters were found to demonstrate significantly different BOLD activity in response to threat cues relative to safe cues. These clusters spanned the right precuneus, right inferior parietal lobule, bilateral cingulate gyrus, and right middle frontal gyrus (Table 3, Fig. 2). Deactivation for safe cues relative to threat cues was observed across the precuneus and the cingulate gyrus. In the precentral and middle frontal gyri, threat cues elicited greater activation.

**Table 3:** Brain regions with significantly different BOLD activation to threat stimuli relative to safe stimuli across participants.

Structure	Vol. μl	HS	MNI coordinates (max peak)			*P_FWEc_*
			x	y	z	
Precuneus, Inferior parietal lobule, Postcentral gyrus	1153	R	28	−50	52	0.003
Cingulate gyrus^a^	456	R	8	16	46	0.016
Precentral gyrus, Middle frontal gyrus	304	R	44	−4	48	0.029

*Note.* While the centre of mass of activity in the cingulate gyrus falls within the right hemisphere, the lateral extent of this cluster was bilateral.

**Figure 3:**
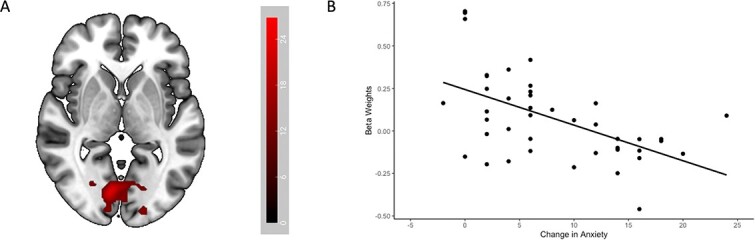
(A) Cluster of activation encompassing the left and right cuneus and lingual gyrus for interaction between the threat versus safe contrast and change in anxiety from baseline to week 6. Activation maps presented at *P* < .001 corrected, with the colour bar representing T-scores. (B) Scatterplot of beta weights for threat versus safe contrast against change in anxiety from baseline to week 6 (positive scores indicate a reduction in anxiety from baseline to week 6). Beta values derived from the highest extent threshold of cluster spanning cuneus and lingual gyrus at MNI coordinates (*x*, *y*, *z*) = −20, −76, 4. Positive beta weights indicate greater activation for threat cues versus safe cues.

Exploratory analyses were conducted to examine sex differences and the effect of change in DASS anxiety from baseline to week 6 on neural responses across treatment groups and cue conditions (threat, safe). No effect of sex on threat versus safe cue responses was found, nor interaction between sex and treatment group. While there was no significant interaction effect between treatment group and change in anxiety, there was an effect of change in anxiety on response to threat versus safe cues in a cluster encompassing the left and right cuneus and lingual gyrus (*P_FWEc_* = .010; peak MNI coordinates: −20, −76, 4). That is, greater reductions in anxiety from baseline to week 6 were associated with a reduced neural response to threat cues relative to safe cues (Fig. 3).

## Discussion

The current study compared the effects of topiramate and naltrexone on fMRI neural activation during anticipatory anxiety in individuals with AUD. Contrary to our hypotheses, there were no differences in neural activation between these treatment groups. Across both groups, deactivation for safe cues relative to threat cues was largely observed across the precuneus, inferior parietal lobule and the cingulate gyrus. In the precentral and middle frontal gyri, threat cues elicited greater activation. Exploratory analyses revealed a significant effect of change in anxiety scores from baseline to week 6 on the response to threat versus safe cues in the cuneus and lingual gyrus, with a greater reduction associated with a lower response to threat cues relative to safe cues. To our knowledge, this is the first study to compare the effect of topiramate and naltrexone on neural activity in patients with AUD during a threat anticipation paradigm.

There is evidence that individuals with AUD display greater deactivation and reduced connectivity in response to threat or stress cues (Seo et al. 2013; Yang et al. 2013; Zakiniaeiz et al. 2017; Wilcox et al. 2020). This has been observed in key emotional processing and anticipatory hubs such as the PCC. In a pharmaco-fMRI study, Wilcox et al. (2020) found no effect of prazosin on anticipatory anxiety in participants with AUD, however, greater deactivation was observed in the PCC and ventromedial prefrontal cortex in response to threat versus safe cues. Greater deactivation in response to threat cues relative to safe cues is purported to reflect inappropriate disengagement in response to stress or over-engagement during low-stress situations (Yang et al. 2013), and has been found to be associated with a greater risk of relapse in treatment-seeking inpatients (Seo et al. 2013). Contrastingly, in the current study we found greater deactivation in response to safe cues relative to threat cues in the precuneus, inferior parietal lobule and cingulate gyrus; a pattern previously observed in healthy controls (Yang et al. 2013). It has been suggested that restoration of brain circuitry following treatment in patients with AUD may be reflected in less deactivation to high threat relative to low threat (Wilcox et al. 2020). Our findings therefore suggest that there may be a normalization of neural activity following treatment with topiramate and naltrexone. Indeed, as reported in the wider trial, participants in both groups reported substantially reduced alcohol consumption at week 6 relative to baseline (Morley et al. 2024). Thus, while topiramate and naltrexone may act on different neurobiological substrates, our demonstration that there were no differences between the medications in response to anticipatory anxiety cues may indicate a comparable impact on the neural circuitries underlying anticipatory anxiety that could be a secondary effect of reduced heavy drinking following treatment.

To this degree, it is worth noting that despite differing biological mechanisms, both topiramate and naltrexone demonstrated an anti-anxiolytic effect in the wider trial, with no group differences observed across the treatment period (Morley et al. 2024). Substantial reductions in anxiety scores were observed in the current sub-study, with participants scoring within the normal range for anxiety by 6 weeks of treatment. Previous work has demonstrated an association between generalized anxiety and anticipatory neural response (Nitschke et al. 2009), whereby individuals with greater anxiety displayed heightened anticipatory activity in the bilateral dorsal amygdala prior to aversive and neutral images. Individuals with elevated anxiety levels have also been found to demonstrate increased activation in the insula (Chua et al. 1999; Simmons et al. 2006) and ACC (Chua et al. 1999) during the anticipation of aversive visual stimuli. The observed similarity in neural activity between topiramate and naltrexone in response to anticipatory cues could thus be due to comparably reduced anxiety at the time of scanning (at weeks 6–8 of treatment). In support of this supposition, we found an effect of change in anxiety from baseline to week 6 on threat versus safe responses in the cuneus and lingual gyrus, such that greater reductions in anxiety scores from baseline to week 6 were associated with a lower response to threat cues relative to safe cues in these regions. The cuneus and lingual gyrus have previously been implicated in fear processing (Carlson et al. 2009; Zhang et al. 2016), and have been found to be engaged during uncertain threat anticipation (Drabant et al. 2011; Michalska et al. 2023). Our finding of an association between generalized anxiety scores and anticipatory neural responses could suggest that the absence of a difference in neural activity between topiramate and naltrexone in response to anticipatory cues may reflect an overall reduction in anxiety symptomatology across the trial. That is, the medications may regulate neural processing of anxiety and threat through the reduction of subjective anxiety levels, including dampening the response to threat cues that may be heightened in individuals with high anxiety levels.

There are strengths and limitations that should be considered together with the current findings. A major strength of the study is that the pharmaco-fMRI neuroimaging approach was conducted within the context of a randomized clinical trial (Grodin and Ray 2019). Further, we employed a well-validated task to evaluate the anticipatory neural response to threat stimuli in an AUD sample (Wilcox et al. 2020; Morley et al. 2021). The absence of baseline scans, however, precluded us from determining changes in neural activity following treatment and clarifying whether the medications normalise or reduce aberrant neural activation in response to anticipatory processing. Given the modest sample size, it is plausible that insufficient statistical power may have limited our ability to detect the presence or absence of a difference between topiramate and naltrexone. Further, the sample size limited our capacity to explore relevant subgroups such as those with co-morbid anxiety disorders (Wilcox et al. 2020). As aforementioned, individuals diagnosed with anxiety disorders display altered anticipatory activity in response to threat cues compared to safe cues (Nitschke et al. 2009). Despite these limitations, this study is the first to compare the effects of topiramate and naltrexone on anticipatory anxiety in patients with AUD. This work serves as a starting point for future investigations into the mechanisms of these medications in the treatment of AUD.

In summary, treatment with topiramate or naltrexone yielded no difference in neural responses to stimuli that elicit anticipatory anxiety in individuals with AUD. Both treatment groups showed greater activation in response to threat cues compared to safe cues in key hubs implicated in threat valence such as the precuneus, inferior parietal lobule, cingulate gyrus and middle frontal gyrus. These results may suggest that both medications have similar effects on the neural circuitry underlying anticipatory anxiety, however, further research is required to elucidate the precise role of topiramate and naltrexone in normalizing anxiety responses in AUD.

## Data Availability

The data underlying this study will be shared upon reasonable request to the corresponding author.
